# Diseases Admitted under the Care of the Physicians of the Westminster Hospital, from the 20th of September to the 20th of October

**Published:** 1799-11

**Authors:** 


*Diseases admitted under the care of the Physicians of the Westminster Hospital, from the 20th of September to the 20th of October.*

| | | | |
|---|---|---|---|
| Fevers - - | 10 | Erythema - - | 2 |
| Scarlatina - - | 2 | Gastrodynia - - | 3 |
| Small-pox - - | 1 | Hooping Cough - | 1 |
| Amenorrhœa - - | 6 | Hæmoptoe - - | 1 |
| Anasarca - - | 5 | Hysteria - - | 1 |
| Ascites - - - | 2 | Impetigo - - | 3 |
| Asthenia - - | 1 | Itch - - - | 3 |
| Asthma - - | 1 | Leucorrhœa - - | 2 |
| Catarrh - - | 1 | Lumbago - - | 2 |
| Cholera - - | 2 | Phthisis - - | 2 |
| Colic - - - | 1 | Pleurodynia - - | 3 |
| Cough - - | 8 | Rheumatism - - | 9 |
| Cephalœa - - | 3 | Struma - - - | 4 |
| Diarrhœa - - | 8 | Vomiting - - - | 2 |
| Dysentery - - | 2 | Worms - - | 2 |
| Enterodynia - - | 1 | | |

The tincture of digitalis, according to Dr. MACLEAN's formula,* has been employed in hæmoptoe and phthisis with evident diminution of the expectoration; but we have not yet seen any case in which it has effected a cure of phthisis.

* Vide Journal, No. vii. p. 122.

